# Multiple myeloma: retrospective assessment of routine thromboprophylaxis and utility of thrombotic risk scores

**DOI:** 10.1016/j.rpth.2024.102571

**Published:** 2024-09-12

**Authors:** Omar Eduardo Fernandez-Vargas, Isabel Amezcua, Beatriz Cabello, Andrea Quintana Martinez, Ramiro Espinoza, Gabriela Cesarman-Maus

**Affiliations:** Department of Hematology, Instituto Nacional de Cancerología, Mexico City, Mexico

**Keywords:** aspirin, multiple myeloma, prophylaxis, risk factors, venous thromboembolism

## Abstract

**Background:**

The high risk of venous thromboembolism (VTE) in multiple myeloma (MM) warrants primary thromboprophylaxis for most patients. Myeloma-specific thrombotic risk scores (TRSs), such as IMPEDE-VTE, SAVED, and PRISM, were developed to improve risk assessment and guide antithrombotic strategies. Their performance is variable and has not yet been tested in Latin America.

**Objectives:**

We aimed to assess the use of primary thromboprophylaxis, the incidence of VTE and bleeding events, and the effectiveness of TRSs in patients with newly diagnosed MM.

**Methods:**

This was a retrospective, single-center study. Cumulative VTE rates and TRS performance were analyzed using survival and receiver operating characteristic curves.

**Results:**

The study included 250 newly diagnosed MM patients; the vast majority (98.6%) received aspirin as thromboprophylaxis. VTE occurred in 8% within the initial 6 months, increasing to 14.8% over a median follow-up of 19 months. High rates of major bleeding (4.8%) and clinically relevant nonmajor bleeding (4.4%) events were documented. A minimal proportion (0.8%, 0.5%, and 1.2%) of patients were classified as low risk by IMPEDE-VTE, PRISM, and SAVED scores, respectively. Only IMPEDE-VTE exhibited a trend for distinguishing between intermediate-risk (7.14%) and high-risk (13.2%) groups (*P* = .09). PRISM and SAVED scores showed limited utility. VTE did not impact survival.

**Conclusion:**

Aspirin as primary thromboprophylaxis carries an unacceptable risk of VTE and bleeding in patients at intermediate or high thrombotic risk. The IMPEDE-VTE score performed best, although without reaching statistical significance. We confirm that VTE does not portend poor overall survival in MM.

## Essentials

• Guidelines recommend thromboprophylaxis for most newly diagnosed multiple myeloma (NDMM) cases.
• Primary thromboprophylaxis with aspirin carries an elevated risk of venous thromboembolism (VTE) and bleeding events.
• Only the IMPEDE-VTE score differentiated risk groups but without statistical significance.
• VTE does not adversely impact survival in patients with NDMM.

## Visual Abstract

**Figure:**
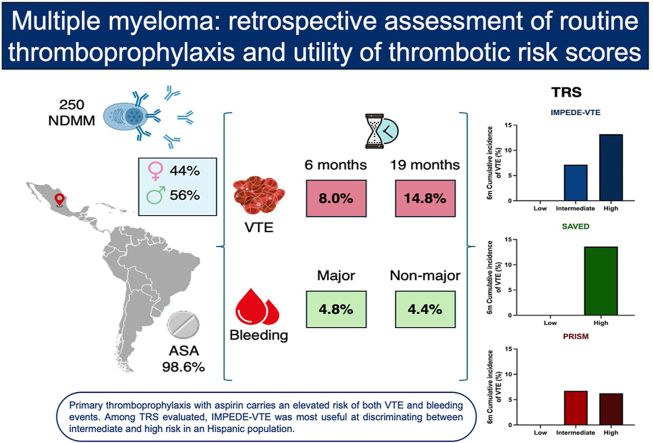

## 1 Introduction

Venous thromboembolism (VTE) is a frequent complication in individuals with newly diagnosed multiple myeloma (NDMM). Despite the use of thromboprophylaxis, the reported rates range between 6.4% and 16.1% with aspirin (ASA), 1.2% and 5% with low-molecular-weight heparins (LMWH), 4% and 8.2% with vitamin K antagonists (VKAs), and recently, 0% and 4.8% with oral factor (F)Xa inhibitors [[1], [2], [3], [4], [5], [6]]. The underlying mechanisms of VTE in multiple myeloma (MM) are multifactorial, primarily driven by factors such as incapacitating fractures and the use of prothrombotic drugs, including immunomodulatory drugs (IMiDs), high-dose dexamethasone (greater than 160 mg/mo), and anthracyclines [7,8]. Interestingly, proteasome inhibitors (PIs) have been reported to confer protection against vascular events [9].

Considering the elevated risk of VTE, current guidelines recommend thromboprophylaxis for all NDMM patients without contraindications [7]. The use of ASA became widespread after a study conducted by Palumbo et al. [3], which prospectively compared LMWH, warfarin, and ASA for primary thromboprophylaxis in patients treated with thalidomide-containing regimens. The rates of VTE with ASA (6.4%) and LMWH (5%) were slightly lower than those with fixed low-dose warfarin (8.2%). Of note, most patients in this study can be retrospectively classified as high-risk by current MM-specific thrombotic risk scores (TRSs) [3].

TRSs specific to MM aim to optimize antithrombotic management. The International Myeloma Working Group (IMWG) criteria, along with the newer Immunomodulatory agent; body Mass Index ≥ 25 kg/m^2^; Pelvic, hip or femur fracture; Erythropoietin stimulating agent; Dexamethasone/Doxorubicin; Asian Ethnicity/Race; VTE history; Tunneled line/central venous catheter; Existing thromboprophylaxis (IMPEDE-VTE) and Prior VTE, Race, IMiD, Surgery, Metaphase Cytogenetics (PRISM) scores, categorize patients into low-, intermediate-, and high-risk categories, while the Surgery, Asian race, VTE history, Eighty age, Dexamethasone (SAVED) score separates IMiD-treated patients into low- and high-risk groups [[10], [11], [12], [13]]. Guidelines suggest that patients at low risk of VTE, as defined by TRSs, receive low-dose ASA (81-325 mg daily), while individuals with intermediate-to-high risk receive LMWH or VKAs [[14], [15], [16]].

Recently published guidelines introduced the option of using fondaparinux 2.5 mg daily or oral FXa inhibitors at prophylactic doses, such as apixaban (2.5 mg twice daily) or rivaroxaban (10 mg daily) [10,14]. Oral Xa inhibitors represent a promising option for thromboprophylaxis in MM, supported mostly by retrospective and a single prospective study [17,18]. Although larger prospective studies are needed to assess the efficacy and safety of oral Xa inhibitors, many treatment centers have already incorporated them into primary strategies for thromboprophylaxis [19,20].

Treatment patterns have been evaluated in MM in Latin America (LATAM) [21,22]; however, data on VTE rates and thromboprophylaxis efficacy are lacking; therefore, the aim of this study was to determine the incidence of VTE and hemorrhagic events with routine thromboprophylaxis to assess the performance of MM-specific TRSs in patients with NDMM, and to evaluate the impact of VTE on overall survival (OS).

## 2 Methods

### 2.1 Study design

This was a descriptive, retrospective study involving the review of electronic medical records from consecutive patients with NDMM cared for at the National Cancer Institute in Mexico, a single tertiary referral cancer center, between January 2016 and June 2022.

### 2.2 Participants

The study population included patients aged 18 years or older with NDMM according to the IMWG criteria. Participants had to have initiated anti-MM therapy with a minimum follow-up period of 3 months. Patients were excluded from the study if they required anticoagulation or antiplatelet agents for reasons other than primary thromboprophylaxis for MM, as well as those patients who had started treatment for MM more than 2 months prior to initiating care at the National Cancer Institute.

### 2.3 Measures

The primary objective was to assess the rate and type of thromboprophylaxis used at our institution. Secondary objectives were to evaluate the incidence of both thrombotic and hemorrhagic events. When clinically suspected, VTE events were confirmed by Doppler ultrasound or computed tomography imaging, performed at the discretion of the treating physician. Incidental pulmonary embolism (PE) was also documented. Arterial thrombotic events were verified using Doppler ultrasound, magnetic resonance imaging, or angio-computed tomography, complemented by additional diagnostic tests such as electrocardiogram and cardiac enzyme measurement, when appropriate. None of the patients had imaging studies done routinely for screening purposes. The severity of hemorrhagic events was classified according to the criteria established by the International Society on Thrombosis and Haemostasis (ISTH) [23]. The third study outcome was the assessment of TRSs’ efficacy in identifying patients with an increased thrombotic risk. The IMPEDE-VTE and SAVED scores were employed for all patients, and the PRISM score was exclusively applied to patients with available metaphase cytogenetic results. Additionally, the mortality rate was determined, and its causes were investigated. The impact of VTE on OS was evaluated with the log-rank test.

### 2.4 Statistical analysis

A descriptive analysis was performed using means and SDs for normally distributed quantitative variables, medians and ranges were used for non-Gaussian distributed quantitative variables, and proportions for qualitative variables. The TRSs were compared using receiver operating characteristic (ROC) curve analysis, determining the area under the curve and 95% CIs for each scale. Differences between groups were assessed through bivariate analysis using the Student's *t*-test or Mann–Whitney U-test for quantitative variables, based on their distribution, and the chi-square test for qualitative variables. For OS and thrombosis-free survival, the Kaplan–Meier method was utilized. We compared the cumulative VTE rates for each risk group according to the different TRSs using the log-rank tests for the entire study duration, as well as for the prespecified length of follow-up validated for each TRS (6 months for IMPEDE-VTE and SAVED, and 6 and 12 months for PRISM). For ROC curve analysis, the TRS scales were treated as ordinal scales, transforming the scale into a series of binary classification problems employing different thresholds and evaluating the positive and negative cases across such different thresholds. The area under the ROC curve (AUC) provides a measurement of the overall discriminatory ability of each TRS scale. All tests were considered statistically significant if they achieved a *P* value of <.05. For statistical analyses, the software packages GraphPad Prism version 9.5.0 and IBM SPSS version 25 were used.

### 2.5 Ethical approval

The protocol underwent review and received approval by the local ethics committee at the Instituto Nacional de Cancerología.

## 3 Results

### 3.1 Patient characteristics

We reviewed 356 consecutive patient records between 2016 and 2022. Of these,106 patients with a median age of 59 years at MM diagnosis were not included for the following reasons: 35 had a follow-up period of less than 3 months from initiation of chemotherapy, these included 13 early deaths and 22 patients who were lost to follow-up; 53 patients had already undergone treatment for more than 2 months prior to the initial evaluation at our institution; 7 patients had incomplete data; 9 patients had plasma cell dyscrasias other than MM; and lastly, 2 patients were on thromboprophylaxis with full anticoagulant doses for atrial fibrillation (1 with apixaban and 1 with rivaroxaban).

A total of 250 patients were included, comprising 110 women and 140 men, with a mean (SD) age of 57.3 ± 11.6 years and a median follow-up of 19 (range, 3-74) months. Table 1 provides a summary of the demographic and clinical characteristics of patients, comparing those with and without confirmed VTE events.

**Table 1:** Baseline demographics for multiple myeloma patients with and without a venous thromboembolism event.

Parameter	W/O VTE (*n* = 213), *n* (%)^a^	W/ VTE (*n* = 37), *n* (%)^a^	*P* value
Age, y (mean ± SD)	57.3 ± 11.8	57.3 ± 11.7	.99
Sex			.86
Female	93 (43.6%)	17 (45%)	
Male	120 (56.3%)	20 (55%)	
Weight, kg	67 (37-132)	67 (49-110)	.64
Height, m	1.58,701,031	1.5,960,103	.61
BMI, kg/m^2^	26.17 (15.69-50.78)	27.08 (19.38-35.29)	.59
ISS stage (*n* = 248)			
Stage I	86 (40.3%)	13 (35.1%)	I vs II, .99
Stage II	62 (29.1%)	9 (24.3%)	I vs III, .30
Stage III	63 (29.5%)	15 (40.54%)	II vs III, .37
R-ISS stage (*n* = 158)^b^			
Stage I	33 (24.4%)	3 (13.0%)	I vs II, .28
Stage II	93 (68.9%)	18 (78.3%)	I vs III, .58
Stage III	9 (6.7%)	2 (8.7%)	II vs III, .99
ASCT^c^			.29
Yes	26 (12.2%)	7 (18.9%)	
No	187 (87.8%)	30 (81.1%)	
CRAB criteria and laboratory characteristics (hypercalcemia, renal insufficiency, anemia, bone lesions)			
Hypercalcemia > 11 mg/L			.38
Yes	42 (19.7%)	10 (27%)	
No	171 (80.3%)	27 (73%)	
Cr > 2 mg/dL			.19
Yes	24 (11.3%)	7 (19%)	
No	189 (88.7%)	30 (81%)	
Anemia^d^			.07
Yes	50 (23.4%)	14 (37.8%)	
No	163 (76.6%)	23 (62.2%)	
Bone lesions (>1)			.48
Yes	197 (92.4%)	36 (97.3%)	
No	16 (7.6%)	1 (2.7%)	
Hgb, g/dL	11.72,776	11.262,798	.31
Leukocytes, ×10^9^/L	6.0 (1.53-61.53)	6.0 (1.6-10.5)	.49
Neutrophils, ×10^9^/L	3.5 (0.9-16.0)	3.3 (0.6-8.4)	.42
Lymphocytes, ×10^9^/L	1.8 (0.1-7.7)	1.9 (0.7-4.7)	.54
Monocytes, ×10^9^/L	0.5 (0-23.1)	0.4 (0.1-1.4)	.51
Platelets, ×10^9^/L	254 (6-696)	251 (55-595)	.98
Cr, mg/dL	0.84 (0.2-26)	1.03 (0.36-11.1)	.08
B2M, mg/L	3.7 (0.6-30.1)	4.2 (1.8-24)	.12
Albumin, g/dL	3.8 (1.5-4.9)	3.9 (1.3-4.5)	.29
Free light chain only			.66
Yes	43 (20.1%)	9 (24.3%)	
No	170 (79.8%)	28 (75.6%)	
Heavy chain involved			IgA vs IgG (*P* = .999)
IgG	113 (53%)	18 (48.6%)	
IgA	55 (25.8%)	9 (24.3%)	
IgM	2 (0.9%)	0	
Biclonal	0	1 (2.7%)	
None (light chain only)	43 (20.1%)	9 (24.3%)	
Light chain involved			.999
Kappa	135 (63.3%)	24 (64.8%)	
Lambda	78 (36.6%)	13 (35.1%)	

ASCT, autologous hematopoietic stem cell transplant; B2M, beta 2 microglobulin; BMI, body mass index; Cr, creatinine; Hgb, hemoglobin; Ig, immunoglobulin; ISS, International Staging System; VTE, venous thromboembolism; W/, with; W/O, without.

a Unless otherwise specified.

b 158 with adequate fluorescence in situ hybridization reports.

c Of the 7 patients with VTE who had ASCT, only 2 occurred after transplant.

d Anemia was defined as a Hgb value of >2 g/dL below the lower limit of normal, or a hemoglobin value < 10 g/dL.

### 3.2 Thromboprophylaxis and thrombotic events

The majority of patients (*n* = 248; 98.6%) received thromboprophylaxis with ASA 81 mg, while only 2 were on apixaban from the start, and 4 patients transitioned from ASA to apixaban as prophylaxis during follow-up. Regarding the rate of thrombosis, 20 (8%) VTE events and 3 (1.2%) arterial thrombosis were confirmed during the initial 6 months of treatment. An additional 8 (3.2%) patients developed VTE between months 7 through 12 and 9 (3.6%) after 12 months of having initiated therapy. These events resulted in a cumulative VTE rate of 14.8% throughout the entire follow-up period. Only 4 thrombotic events were asymptomatic and detected incidentally when imaging studies were carried out for different reasons.

By site of thrombosis, 31 events involved the lower extremities, and 3 developed PE; 2 patients presented with superficial venous thrombosis, and 1 with temporal vein thrombosis. Patients with confirmed thrombosis were treated with therapeutic anticoagulation using standard dosing of oral FXa inhibitors. Except for 2 patients, all individuals were using thromboprophylaxis at the time of VTE; 1 patient was taking apixaban, while all others were on ASA.

### 3.3 Hemorrhagic events

A total of 14 (4.8%) major bleeding (MB) events were documented in 12 patients; 7 were gastrointestinal (GI), 5 involved the central nervous system, and 2 originated from extramedullary plasmacytomas. Nine patients were on thromboprophylaxis with ASA at the time of MB, and 3 were on therapeutic doses of anticoagulants for the treatment of VTE (1 each with either acenocoumarol, apixaban, or rivaroxaban). Of patients with MB events, 11 required packed red blood cell transfusions with a median of 3 (range, 2-12) units.

Additionally, 11 (4.4%) patients had clinically relevant non-MB (CRNMB) events, all associated with low-dose ASA. Of these, 6 were GI bleeds, 1 was oral mucosal bleeding, and 4 were epistaxis. Two of the patients with CRNMB also had a history of an MB event, and 4 patients were on therapeutic doses of oral FXa inhibitors for the treatment of VTE.

### 3.4 TRS evaluation

**Figure 1:**
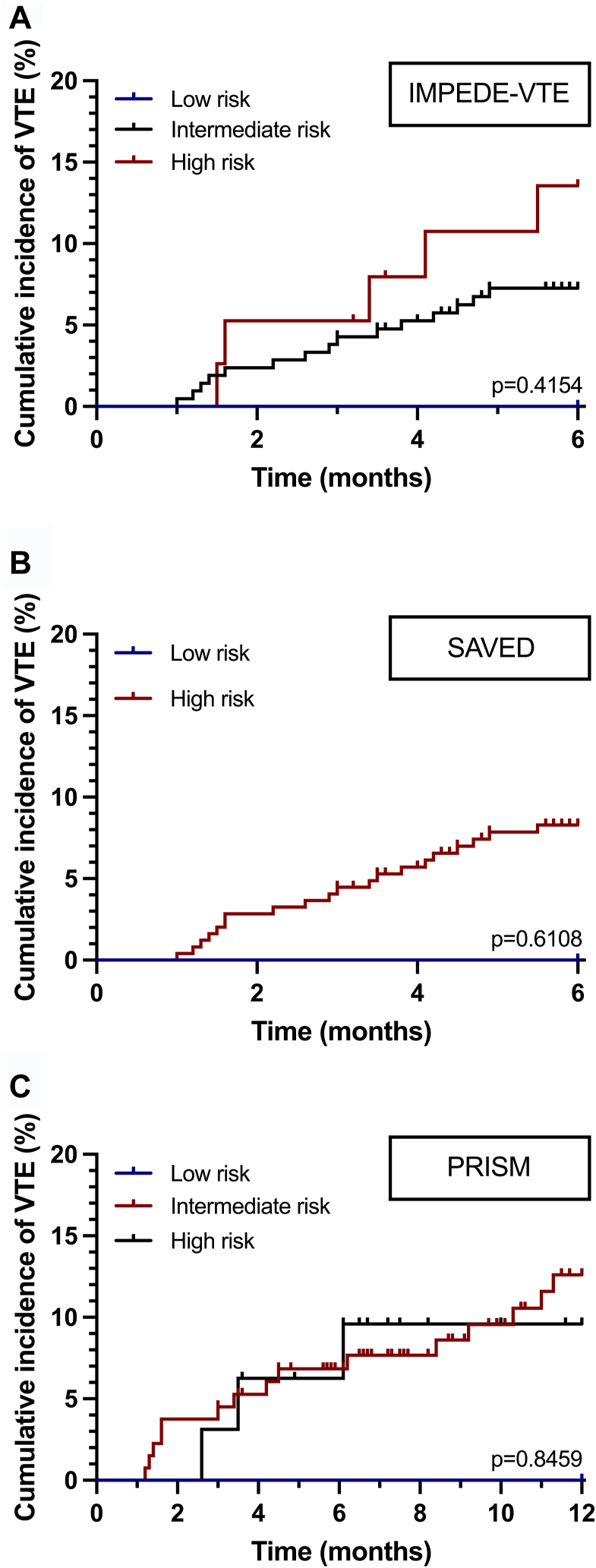
Cumulative incidence of venous thromboembolism (VTE) by thrombotic risk score (TRS) risk stratification. Kaplan–Meier curves for 6-month cumulative VTE for IMPEDE-VTE (A), SAVED (B), and 12 months cumulative VTE for PRISM (C).

We assessed the utility of TRSs in detecting individuals at higher risk of developing VTE; however, only 2 (0.8%), 2 (0.5%), and 3 (1.2%) patients were classified as low-risk according to IMPEDE-VTE, PRISM, and SAVED scores, respectively (Table 2). When employing the IMPEDE-VTE score, the incidence of thrombosis at 6 months in the high-risk group (13.2%) nearly doubled that of patients with intermediate-risk (7.14%; Figure 1A), with an AUC by ROC analysis of 61.5% (95% CI, 48.8%-0.74%; *P* = .09).

**Table 2:** Performance of thrombotic risk scores.

TRS	Risk group	Group distribution, *n* (%)	6-mo VTE rate, %	*P* value
IMPEDE-VTE	Low	2 (0.8%)	0%	.41 (ns)
	Intermediate	210 (84%)	7.14%	
	High	38 (15.2%)	13.2%	
SAVED	Low	3 (1.2%)	0%	.999 (ns)
	High	247 (98.8%)	8.09%	
PRISM	Low	2 (1.3%)	0%	.93 (ns)
	Intermediate	122 (78.2%)	6.7%	
	High	32 (20.5%)	6.3%	

Ns, not significant; TRS, thrombotic risk score; VTE, venous thromboembolism.

The SAVED score is meant to distinguish patients only between low- and high-risk categories, with 99.5% of our patients falling into the high-risk group, with an 8.1% incidence of VTE at 6 months (Figure 1B), and an AUC by ROC analysis of 50.4% (95% CI, 37.4%-63.5%; *P* = .94).

**Figure 2:**
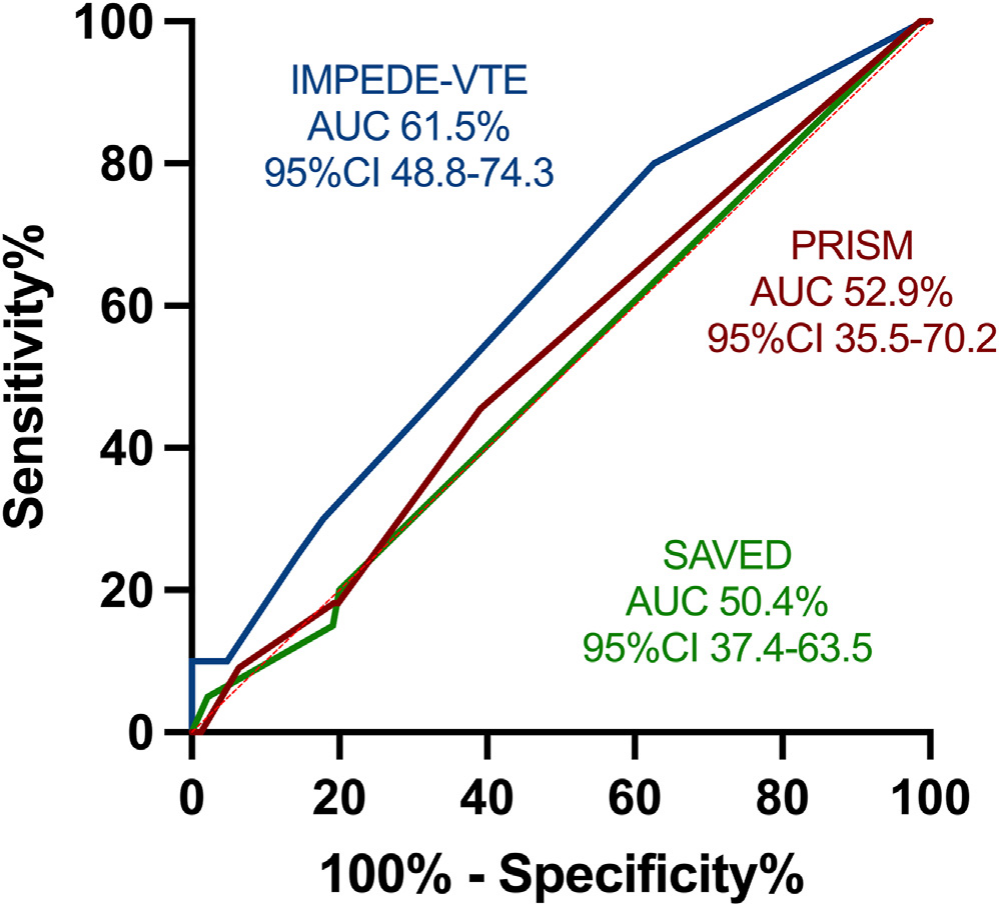
Thrombotic risk score performance evaluation. Sensitivity and specificity were determined using receiver operating characteristic curve analysis at 6 months for the 3 scores being evaluated. Briefly, scales were transformed into a series of binary classification problems, with the area under the curve employed as a measure of overall thrombotic risk score performance.

Lastly, the PRISM score, which was designed to evaluate thrombotic risk at 6 and 12 months, was applied to 167 patients in whom metaphase cytogenetic results were available. Of these latter patients, 133/167 (79.6%) were classified as intermediate-risk and 32/167 (19.1%) as high-risk. The cumulative VTE incidence was 11.3% and 9.4% for the intermediate-risk and high-risk groups, respectively (Figure 1C). The AUC for ROC analysis at 6 months was 0.5286 (95% CI, 0.36-0.70; *P* = .75), and at 12 months, it was 0.5246 (95% CI, 0.38-0.67; *P* = .73). ROC curve analysis for IMPEDE-VTE, SAVED, and PRISM are summarized in Figure 2.

**Supplementary Figure S1:**
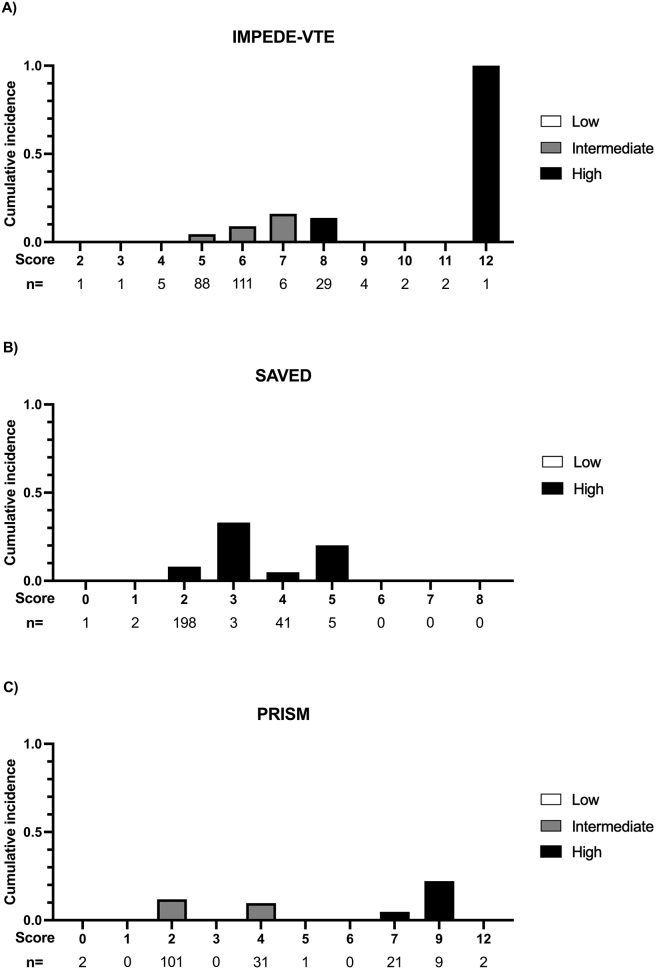
Cumulative VTE incidence by TRS score. Cumulative incidence of VTE over different time frames, based on points scored by each validated TRS: (A) IMPEDE-VTE for 6 months, (B) SAVED for 6 months, and (C) PRISM for 12 months.

The stratification of risk groups did not agree between the 3 TRSs. The 2 patients classified as low-risk by IMPEDE-VTE were not the same as those with low-risk by SAVED or PRISM. Moreover, Cohen’s kappa coefficient for reliability between high- and intermediate-risk patients for IMPEDE-VTE and PRISM was 0.185 (95% CI, 0.007-0.36), suggesting only a slight agreement. Moreover, Supplementary Figure S1 shows the cumulative VTE incidence, divided by scored points and time frames validated for each TRS.

Multivariate analysis was conducted to explore the association between the evaluated items defined in the different scores (Supplementary Table), consistently showing a low yield for individual items for each TRS.

### 3.5 Anti-MM therapy

Patients with NDMM were administered first-line treatment with various agents, including dexamethasone (*n* = 234; 93.6%), IMiDs (*n* = 248; 99.2%), PIs (*n* = 98; 39.2%), daratumumab (*n* = 16; 6.4%), and doxorubicin (*n* = 7; 2.8%). By regime, triplets were the most commonly used, with the combination of an IMiD, cyclophosphamide, and dexamethasone being the most frequent (*n* = 152). Quadruplets were also commonly used, with the combinations of IMiDs, PIs, cyclophosphamide, and dexamethasone (*n* = 73), as well as IMiDs, PIs, anti-CD38, and cyclophosphamide (*n* = 16). Another regimen, consisting of cyclophosphamide, doxorubicin, dexamethasone, and IMiDs, was used in 7 patients. Lastly, a doublet regimen of carfilzomib and dexamethasone was used in 2 patients.

There were no patients treated with erythropoietin stimulating agents, and only 3 patients received lenalidomide, while the rest had thalidomide (*n* = 245). A total of 33 (13.2%) patients underwent consolidation with autologous hematopoietic stem cell transplant, of whom 7 experienced VTE events, but only 2 of these events occurred after autologous hematopoietic stem cell transplant.

### 3.6 Survival analysis

With a median follow-up time of 19 months (range, 3-74), the median OS was reached at 62 months. Concerning mortality, 64 deaths were recorded at a rate of 25.6%. The primary causes of death were infectious complications (*n* = 33; 51.5%), disease progression (*n* = 5; 7.8%), cardiovascular events (*n* = 5; 7.8%), including 1 myocardial infarction and 1 sudden death, and hemorrhagic events (*n* = 3; 4.6%). Four (6.3%) patients died from second malignancies, while in 14 (21.8%), the cause of death was not documented.

**Figure 3:**
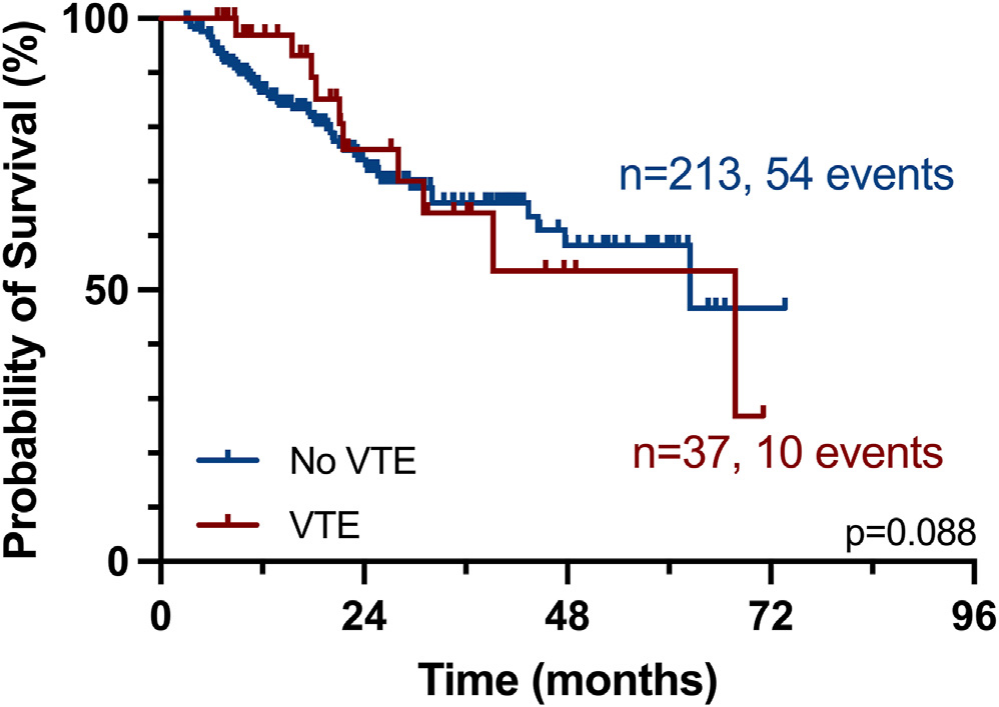
Overall survival and impact of venous thromboembolism (VTE) events. Overall survival as determined by the Kaplan–Meier method for patients with (red) and without VTE (blue).

Among the hemorrhagic events, one patient experienced GI bleeding, another had a subdural hematoma, and a third patient suffered an ischemic stroke with hemorrhagic transformation and subsequent intracranial hypertension. All fatal hemorrhagic events were associated with the use of ASA. Notably, our data show that OS for the entire cohort was not adversely affected by VTE (Figure 3).

## 4 Discussion

### 4.1 Key results

All patients treated for MM received thromboprophylaxis, the vast majority (98.9%) with ASA and only 1.1% with apixaban. Despite the generalized administration of thromboprophylaxis, with a median follow-up of 19 months, 14.8% of the patients developed VTE events. Eight percent of patients developed a thrombotic event within the first 6 months, with the risk remaining significant at >3% thereafter. Most of the events were symptomatic, including 3 cases of PE. Arterial events were rare, occurring in only 3 patients. In terms of safety, there were 14 (4.8%) MB events with 3 associated deaths. GI bleeding was the most common site of both MB and CRNMB.

Concerning the performance of the TRSs, only a minority of our patients fell into the low-risk category (0.8%-1.2%). This was attributed to the prevalent use of high doses of dexamethasone (>160 mg per month) and IMiD-based therapy in most cases. Interestingly, the patients classified as low-risk by the different scales did not coincide. IMPEDE-VTE was the only score to successfully differentiate between intermediate-risk (7.14%) and high-risk (13.2%) of VTE at 6 months, although it did not reach statistical significance (*P* = .09). In contrast, the PRISM score failed to distinguish between high-risk and intermediate-risk of VTE at 12 months (9.4% vs 11.3%, respectively), while the SAVED score did not prove useful, as almost all patients were classified in the high-risk group. Notably, there was no observed difference in OS for patients who developed VTE compared with those who did not.

### 4.2 Interpretation

Given the high risk of VTE associated with the treatment of NDMM, the IMWG issued a recommendation to administer thromboprophylaxis to all MM patients based on thrombotic risk [10]. In a real-world evidence study of 306 patients with MM, the effectiveness of IMWG guidelines was assessed, revealing that only 19% (56 patients; 22 classified as low-risk and 34 as high-risk) received appropriate thromboprophylaxis in accordance with the guideline, concluding that adherence to the recommendations is low and posing challenges in determining their effectiveness [24]. The phase 3 Myeloma XI trial incorporated the IMWG guidelines, showing a substantial increase in the adoption of thromboprophylaxis compared with the Myeloma IX trial (80.5% vs 22.3%). This was accompanied by a decrease in VTE incidence for identical therapeutic regimens: CTD (cyclophosphamide, thalidomide, and dexamethasone; 13.2% vs 16.1%) and attenuated CTD (10.7% vs 16%). Despite the implementation of IMWG-guided thromboprophylaxis, the incidence of thrombosis remained high [25]. More recent prospective randomized studies with currently preferred regimens such as RVd (lenalidomide, bortezomib, and dexamethasone) ± daratumumab (GRIFFIN study) report a high rate of thrombosis (10.1% with and 15.7% without daratumumab) due partly to suboptimal thromboprophylaxis [26].

The widespread use of ASA as a thromboprophylaxis measure for MM began after a phase 3 study by Palumbo et al. [3], which demonstrated efficacy comparable with LMWH and VKAs, coupled with its low cost, ease of administration, and lack of monitoring requirements. Nevertheless, thrombotic risk remained significant for all study arms (5%-8.2%) [3]. The thrombotic risk, despite prophylaxis, is highly dependent on the chemotherapy regimen. In a clinical trial comparing lenalidomide plus high-dose dexamethasone with and without ASA, the incidence of VTE was 14% in the ASA group compared with 23% for the non-ASA group, showing partial efficacy but high residual risk [8]. In our study, despite thromboprophylaxis with ASA, the incidence of VTE persisted (14.8%). This could in part be due to the fact that the majority of our patients fell within an intermediate-to-high thrombotic risk profile. Yet, a retrospective study by Piedra et al. [4], in which 85.7% of patients on ASA were deemed low thrombotic risk using the SAVED score, reported a 9.8% rate of thrombosis and up to 16.1% when the regimen used was KRD (carfilzomib, lenalidomide, and dexamethasone). In fact, most VTE events for all patients in this study occurred in the low-risk category [4]. The latter suggests that ASA may not be effective even in patients determined to be at low risk by current TRSs. Furthermore, in our study, the occurrence of bleeding on ASA – as defined by the ISTH criteria – was unduly high, with 4.8% MB and 4.4% CRNMB. It is worth mentioning that in published retrospective and prospective studies using oral anti-Xa inhibitors, the incidence for both thrombosis and bleeding seems to be lower than that reported with ASA [4,6,17], which begs the question of whether we should be using oral anti-Xa inhibitors for most patients, regardless of their risk profile.

In reference to thrombotic risk estimation, 3 recently published TRSs have been developed to identify patients at increased risk with the aim of aiding in safer and personalized prophylactic strategies. These include the IMPEDE-VTE, SAVED, and PRISM scores. All consider a prior history of VTE and race. The IMPEDE-VTE score takes into account the use of antiplatelet or anticoagulant agents, with the risk of thrombotic events decreasing based on the specific antithrombotic agent employed. Amerindians are known to have a lower thrombotic risk, just above that of people of Asian descent [27]; however, in our analysis, it was not possible to assess the impact of race due to insufficient representation of patients of Asian or African descent. Nonetheless, other research groups have clearly established the association between race and thrombotic risk [3,10,25,28].

ROC curve analyses showed that IMPEDE-VTE was the only score that distinguished between intermediate- and high-risk patients (VTE 7.2% vs 13.2%); however, none of the TRSs reached statistical significance. In other studies, IMPEDE-VTE showed utility in an external validation cohort, in which 38.7%, 38.9%, and 22.4% of the patients were categorized as low-, intermediate-, and high-risk, with an incidence of VTE of 1.1%, 5.7%, and 11.8%, respectively. Their predictive value was similar to that published by Sanfilippo et al. [28] (AUC-ROC, 0.70; 95% CI, 0.60-0-80); however, only 25% of patients in this validation cohort had received an IMiD [29]. We were not able to detect patients at low risk of thrombosis by any TRS, a finding consistent with other publications [4].

Regarding the PRISM score, its applicability in LATAM is limited due to the less widespread availability of cytogenetic studies, as evidenced by the Hemato-Oncology Latin America Observational Registry study, where only 20% of the population had access to this type of testing [22]. The study by Chakraborty et al. [13] reported an association between abnormal metaphase cytogenetics and risk of VTE, but not with poor prognosis cytogenetic alterations by fluorescence in situ hybridization [del(17p), t(4;14), t(14;16), t(14;20)] [[30], [31], [32]]. In our study, abnormal metaphase cytogenetics were not associated with VTE (*P* = .42). In a retrospective study by Sekar et al. [33], the PRISM score was externally validated, suggesting that abnormal metaphase cytogenetics could be a risk factor for VTE in MM patients. However, abnormal metaphases were detected in only 13% of the study's population. Furthermore, the study revealed considerable variability in primary thromboprophylaxis, with only 53.8% of patients receiving ASA [33].

Finally, concerning the impact of VTE on OS, in both the Myeloma IX and XI studies, there was no clear difference in OS in patients with or without VTE (Myeloma IX adjusted hazard ratio [aHR], 0.87; Myeloma XI aHR, 0.90). However, in the Myeloma XI trial, there was an elevated risk of mortality in patients who developed arterial thrombosis (aHR, 1.53) [25]. In contrast, a trial conducted by Schoen et al. [34] did find a higher 6-month mortality among MM patients who experienced VTE events. This risk was further elevated in patients receiving lenalidomide, with an aHR of 2.31 [34]. In our cohort, VTE did not have a detrimental effect on OS for the entire cohort, though we cannot rule out the occurrence of an undetected fatal PE.

### 4.3 Implications

Concerning the practical implications of our findings, the largest MM study in LATAM to date, by the Grupo de Estudio Latino Americano de Mieloma Múltiple, revealed that NDMM is primarily treated with regimens containing high-dose dexamethasones such as CyBorD (cyclophosphamide/bortezomib/dexamethasone), CTD (cyclophosphamide/thalidomide/dexamethasone), and VTD (bortezomib/thalidomide/dexamethasone). This population had a relatively younger median age at diagnosis, similar to our study (54 vs 57 years). Additionally, approximately 50% of patients received IMiDs. This stratifies most patients into intermediate or high thrombotic risk using current TRS [21]. These treatment patterns closely resemble those observed in our study, albeit with a higher proportion of bortezomib use, suggesting our results may be applicable to countries in LATAM.

### 4.4 Limitations and strengths

Our study has several limitations, mainly its retrospective design and single-center setting. Another constraint was risk stratification, with most patients categorized as intermediate- and high-risk, largely due to the use of high-dose dexamethasone and IMiDs. Consequently, we lack a substantial sample of low-risk patients to evaluate the efficacy of ASA in this subgroup. Moreover, the analysis of race as a clinical predictor of VTE was not feasible, as our population lacked individuals of Asian or African descent. However, the strengths of our study include a comprehensive assessment of different TRSs, including TRSs that consider cytogenetic abnormalities like PRISM in a sizable and mostly homogeneous NDMM cohort receiving thromboprophylaxis with ASA.

## 5 Conclusions

Our current study presents a comprehensive evaluation of the use of routine thromboprophylaxis, its efficacy and safety, and the performance of TRSs in patients with NDMM. During the study period, the vast majority of patients received ASA as primary prophylaxis. The use of regimens with IMiDs and high-dose dexamethasone was the main reason for the stratification of all but a handful of patients into intermediate or high thrombotic risk by different TRSs. We found both thrombotic and bleeding rates to be unacceptably high using ASA. Other authors have found that even in patients stratified into low-risk categories, the use of ASA is not sufficiently protective or safe. This suggests that patients with NDMM without an elevated bleeding risk may benefit from an alternative antithrombotic strategy, independently of the thrombotic risk category.

We found shortcomings of current TRSs for accurately identifying patients at increased thrombotic risk. Only IMPEDE-VTE showed a trend in differentiating patients at intermediate and high thrombotic risk, yet the incidence of thrombosis is high enough in both groups to require a change in prophylactic strategy.

The limitations of our study include its retrospective design, single-center setting, and predominance of intermediate- and high-risk patients, which may affect how broadly applicable our findings are. TRSs may perform better when more patients with low thrombotic risk are included, yet these latter patients are probably not being referred to our center. Nevertheless, the study provides valuable insights into the safety and effectiveness of ASA in a cohort where all patients were receiving thromboprophylaxis. Additionally, it also provides information on the challenges and opportunities for improving thromboprophylaxis and evaluates the usefulness of all 3 major MM TRSs.
