# Supplementary material for: Multiple myeloma: retrospective assessment of routine thromboprophylaxis and utility of thrombotic risk scores
Source: Res Pract Thromb Haemost. 2024 Sep 12;8(7):102571. doi: 10.1016/j.rpth.2024.102571 (PMC11564956; doi:10.1016/j.rpth.2024.102571)
Supplement: Supplementary Table [file mmc1.docx]

**Supplementary Table.** Multivariate analysis of items evaluated in IMPEDE-VTE, SAVED and PRISM scores at a 6-month cut-off.

| **RTS** | **Item** | **Patient distribution** | **6-month cut-off** | |
| --- | --- | --- | --- | --- |
|  |  |  | **Cox proportional HR, HR (95%CI)** | **p value** |
| **IMPEDE-VTE** | IMiD use | 248/250 | N.E. | N.E. |
|  | BMI ≥25 kg/m^2^ | 147/250 | 2.019 (0.713-5.714) | 0.186 |
|  | Pelvic, hip or femur fracture | 18/250 | 1.295 (0.285-5.881) | 0.738 |
|  | Use of ESAs | 16/250 | 2.434 (0.292-20.292) | 0.411 |
|  | Doxorubicin | 6/250 | 5.247 (0.993-27.736) | 0.051 |
|  | Dexamethasone High-dose | 248/250 | N.E. | N.E. |
|  | Dexamethasone Low-dose | 2/250 | N.E. | N.E. |
|  | Ethnicity: Asian/Pacific Islander |  |  |  |
|  | History of VTE before MM | 5/250 | 3.309 (0.391-28.007) | 0.272 |
|  | Tunneled/central venous line | 17/250 | 2.589 (0.503-13.328) | 0.255 |
|  | Therapeutic LMW or warfarin |  |  |  |
|  | Prophylactic LMWH or aspirin | 243/250 | 0.691 (.085-5.639) | 0.730 |
| **SAVED** | Surgery (within 90 days) | 43/250 | 2.016 (0.46-8.83) | 0.352 |
|  | Asian race |  |  |  |
|  | History of VTE | 5/250 | 2.999 (0.398-22.58) | 0.286 |
|  | Age ≥80 years | 5/250 | 3.048 (0.4-23.221) | 0.282 |
|  | Dexamethasone High-dose | 248/250 | N.E. | N.E. |
|  | Dexamethasone Low-dose | 2/250 | N.E. | N.E. |
| **PRISM** | Prior history of VTE | 2/167 | N.E. | N.E. |
|  | Race: Black |  |  |  |
|  | IMiD use | 165/167 | N.E. | N.E. |
|  | Surgery (within 90 days) | 31/167 | 1.068 (0.230-4.95) | 0.933 |
|  | Abnormal metaphase cytogenetics | 44/167 | 0.608 (0.178-2.078) | 0.427 |

Proportional hazards analysis of items evaluated in IMPEDE-VTE, SAVED and PRISM scores at a 6-month cut-off.
